# Longitudinal Association of Perceived Stress with Cerebrospinal Fluid Alzheimer's Disease Biomarkers in a Biracial Cohort

**DOI:** 10.1002/alz.093384

**Published:** 2025-01-09

**Authors:** Antoine R Trammell, Felicia Goldstein, James J. Lah, Monica W Parker, Crystal P Davis, Cornelya D Dorbin, Sabria Saleh, Elizabeth Murphy‐Tchivzhel, Alecia Michella Rose, Amber Osborne, Adiva Ahmad, Jahmila Al‐Amin, Ihab Hajjar

**Affiliations:** ^1^ Emory University Goizueta Alzheimer's Disease Research Center, Atlanta, GA USA; ^2^ Emory University School of Medicine, Atlanta, GA USA; ^3^ Emory University, Atlanta, GA USA; ^4^ UT Southwestern Medical Center, Dallas, TX USA

## Abstract

**Background:**

African American (AA) persons have a higher Alzheimer's disease (AD) prevalence and report more perceived stress than White persons. Our previous cross‐sectional study (JAD, 2020, 77:843‐853) demonstrated an association between self‐reported stress levels and cerebrospinal fluid (CSF) AD biomarkers. Using a biracial cohort, the current study investigates the association between stress and longitudinal CSF AD biomarkers over time.

**Method:**

We analyzed data from a biracial cohort of community‐dwelling adults 50 years or older who completed baseline, Year 1, and Year 2 visits at the Emory Goizueta Alzheimer’s Disease Research Center. Participants underwent a lumbar puncture and cognitive testing and completed the perceived stress scale (PSS) questionnaire at each visit. We created 4 groups by self‐reported race (AA vs. White) and cognitive classification (normal vs. impaired). Using ANOVA or chi‐square, we compared baseline characteristics, cognitive classification status, and CSF AD biomarkers in the 4 groups (white/normal, white/impaired, AA/normal, and AA/impaired). Next, we tested associations between baseline PSS scores (predictor) and CSF AD biomarkers (outcome) over time using mixed models adjusted for factors confounding racial differences in AD. The significance level for all tests was p < 0.05.

**Result:**

Data for 583 participants were analyzed (mean age:64.2; AA:49.7%; female:61.1%; impaired:68.6%). The AA participants were younger (63.6 vs. 67.7, p=.004) than white participants and had less neuropathology (Aβ42: 278 vs. 249, p<.0001; T‐tau: 47.9 vs. 74.7, p<.0001; P‐tau: 16 vs. 23.3, p<.0001; TAR: .21 vs. .37, p<.0001; PTAR: .07 vs. .11, p<.0001). AA/impaired participants reported higher stress (PSS=23.1, p=.04) than other groups. Regression, after adjusting for age, sex, education, BMI, and vascular comorbidities, showed higher baseline PSS scores were associated with higher P‐tau (F=8.22, p=.008) and PTAR (F=9.29, p=.005) over time in AA/impaired participants.

**Conclusion:**

Higher baseline PSS scores were associated with Tau accumulation in the AA/impaired group over time. These results confirm previous findings and further suggest a biological connection underlying the stress‐AD link and its racial disparity. Future work exploring potential mechanisms is needed.

